# Minimally invasive surgery or stenting for left anterior descending artery disease – *meta*-analysis

**DOI:** 10.1016/j.ijcha.2022.101046

**Published:** 2022-05-10

**Authors:** Monica. Gianoli, Anne R. de Jong, Kirolos A. Jacob, Hanae F. Namba, Niels P. van der Kaaij, Pim van der Harst, Willem J.L Suyker

**Affiliations:** a Department of Cardiothoracic Surgery, University Medical Center Utrecht, Utrecht, the Netherlands; b Department of Cardiology, University Medical Center Utrecht, Utrecht, the Netherlands

**Keywords:** Minimally invasive direct coronary artery bypass, Percutaneous coronary intervention, Proximal LAD lesion, Meta-analysis, BMS, bare metal stent, CABG, coronary artery bypass grafting, CI, confidence interval, CVA, cerebrovascular accident, DES, drug eluting stent, LAD, left anterior descending, LITA, left internal thoracic artery, (RA)-MIDCAB, (robotic assisted) minimally invasive direct coronary artery bypass, MAC(C)E, Major Adverse Cardiac (and Cerebrovascular) Events, MI, myocardial infarction, NNT, number needed to treat, PCI, percutaneous coronary intervention, PRISMA, Preferred Reporting Items for Systematic Reviews and Meta-Analyses, RCT, randomized controlled trial, RR, risk ratio, rTVR, repeat target vessel revascularization

## Abstract

Minimally invasive direct coronary artery bypass (MIDCAB) surgery and percutaneous coronary intervention (PCI) are both well-established minimally invasive revascularization strategies in patients with proximal left anterior descending (LAD) lesions. We aimed to evaluate the 20-years’ experience by performing a systematic review and *meta*-analysis comparing MIDCAB versus PCI in adults with proximal LAD disease. We searched MEDLINE, EMBASE and Cochrane on October 1st, 2021 for articles published in the year 2000 or later. The primary outcome was all-cause mortality. Secondary outcomes included cardiac mortality, repeat target vessel revascularization (rTVR), myocardial infarction (MI), and cerebrovascular accident (CVA). Outcomes were analysed at short-term, mid-term, and long-term follow-up. Random effects *meta*-analyses were performed. Events were compared using risk ratios (RR) with 95% confidence intervals (CI). Our search yielded 17 studies pooling 3847 patients. At short-term follow-up, cardiac mortality was higher with MIDCAB than with PCI (RR 7.30, 95% CI: 1.38 to 38.61). At long-term follow-up, MIDCAB showed a decrease in all-cause mortality (RR 0.66, 95% CI: 0.46 to 0.93). MIDCAB showed a decrease in rTVR at mid-term follow-up (RR 0.16, 95% CI: 0.11 to 0.23) and at long-term follow-up (RR 0.25, 95% CI: 0.17 to 0.38). MI and CVA comparisons were not significant. In conclusion, in patients with proximal LAD lesions, MIDCAB showed a higher short-term mortality in the RCTs, but the cohort studies suggested a lower all-cause mortality at long-term follow-up. We confirm a decreased rTVR at mid-term follow-up in the RCTs and long-term follow-up in the cohort studies.

## 1 Introduction

The most recent European Guidelines for myocardial revascularization recommend both coronary artery bypass surgery (CABG) and percutaneous coronary intervention (PCI) for patients with isolated proximal left anterior descending (LAD) disease [1], [2], [3], [4], [5], [6], [7], [8], [9].

In favor of PCI is the less invasive nature of the treatment, while in favor of CABG is the long-term survival benefit offered by the left internal thoracic artery (LITA) to LAD and a decrease in the occurrence of repeat revascularization [10], [11], [12], [13]. Over the past two decades there has been an increased adoption of a minimally invasive direct coronary artery bypass (MIDCAB) strategy to perform the LITA-LAD conduit through a small left thoracotomy. It has been shown that MIDCAB has a similar safety and efficacy profile when compared to conventional CABG [4], [8], [14], [15]. However, it is not clear whether the long-term survival benefit that has been demonstrated with conventional CABG also applies to MIDCAB when compared to PCI and MIDCAB is currently not included in the ESC/EACTS guidelines for revascularization [1].

In this *meta*-analysis, we aimed to aggregate and critically evaluate the best evidence from the past 20 years on long-term outcomes after MIDCAB or PCI in adults with isolated proximal LAD disease.

## 2 Materials and methods

### 2.1 Search strategy

We queried MEDLINE via Pubmed, EMBASE and the Cochrane database on October 1st, 2021, using variations and synonyms of the search terms: minimally invasive direct coronary artery bypass surgery, percutaneous coronary intervention and proximal left anterior descending artery lesions (Appendix 1 for full search strings). The review was performed in accordance with the Preferred Reporting Items for Systematic Reviews and Meta-Analyses (PRISMA) guidelines and aimed to find all published reports comparing MIDCAB and PCI as a revascularization strategy for proximal LAD lesions in adults and was performed in duplicate by two researchers (MG and ARJ) [16]. We also performed a cross-reference check.

### 2.2 Inclusion and exclusion criteria

Cohort studies and randomized controlled trials (RCT) comparing the treatment of adult patients with isolated proximal LAD lesion who underwent MIDCAB or PCI as the primary procedure were included. For inclusion the studies had to be written in English, reporting original data and published in or after the year 2000. For inclusion at least one of the following outcomes of interest had to be reported: of all cause and cardiac mortality, repeat target vessel revascularization (rTVR), myocardial infarction (MI) and cerebrovascular accident (CVA). We excluded studies reporting non isolated LAD lesion treatment, or papers without definition of primary intervention strategy. Papers including a cohort of patients who underwent a primary full sternotomy, were excluded. We excluded papers reporting no original data or papers, without definition of primary intervention strategy and/or the outcomes of interest. Additionally, papers were assessed for their quality using the Risk of Bias 2 (RoB 2) Cochrane tool for randomized trials, whereas the ROBINS-I tool was used for cohort studies [17], [18]. Articles with a high risk of bias were excluded from the analysis.

### 2.3 Data extraction

After the search was performed, two independent reviewers (MG and ARJ) reviewed all articles. Discrepancies and were addressed and solved by a third reviewer (KJ). Data extractions were performed independently by three reviewers (MG, ARJ and HFN). When the same author published multiple studies we extracted patients’ characteristics from the first study and outcomes of interest at subsequent follow-ups from later studies. When two studies by the same institution reported the same outcomes at similar follow-up periods, we included either the higher quality or the most informative publication. Articles assessment was performed with the Cochrane Risk of Bias tool by the three aforementioned reviewers (Appendix 2).

### 2.4 Outcomes

The primary outcome was all-cause mortality at three different timeframes: < 30 days (short-term), 30 days – 1 year (mid-term), and > 3 years (long-term) of follow-up. All-cause mortality was defined as any cause of death.

Secondary outcomes were cardiac mortality, repeat target vessel revascularization (rTVR), myocardial infarction (MI) and cerebrovascular accident (CVA) at < 30 days, 30 days – 1 year, and > 3 years of follow-up. Cardiac mortality was defined as a primary cardiac cause of death. rTVR was defined as repeat revascularization of the target vessel from the original procedure. MI was defined following the definition of the original article. CVA did not include transient ischemic attacks.

### 2.5 Statistical analysis

Statistical analyses were performed using Review Manager (Version 5.3.5 Copenhagen: The Nordic Cochrane Centre, The Cochrane Collaboration, 2014) and the statistical program R (version 4.0.3. 2020 The R Foundation for Statistical Computing). We used random effects models (Mantel-Haenszel method) instead of fixed effects for a more robust and conservative risk ratio (RR). The RR was calculated for categorical variables as the effect estimate for all outcomes. The results were presented as a forest plot, depicting the individual RR from each study as well as the overall composite effect estimate. An RR with its 95% confidence interval (95% CI) <1 would favor MIDCAB. In the inverse weighted model, each study contributed to a percentage of the final pooled estimate, and was presented in each forest plot under the column of weight [19]. According to Bate’s correction, 0.1 was added to each cell of the two-by-two table in case the study or control arm had zero events [20]. We calculated the risk difference with the number needed to treat (NNT).

The *I^2^* statistic and its corresponding *p-*value were calculated to test for heterogeneity. We additionally re-analyzed the data using fixed-effects models. All data were stratified by study design at short-term (<30 days follow-up), mid-term (30 days – 1 year), and long-term (>3 years) follow-up. Publication bias was assessed using visual inspection of the contour-enhanced funnel plot symmetry and with Egger’s test. A *p*-value of 0.05 or less was considered statistically significant.

## 3 Results

### 3.1 Selection and characteristics of included studies

**Fig. 1:**
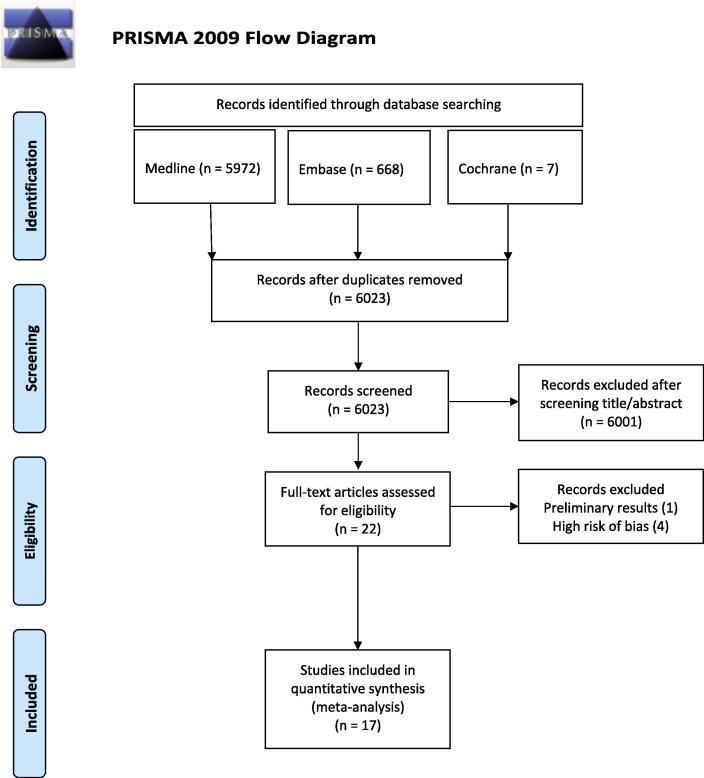
Flow diagram of selected studies.

The literature search yielded a total of 6647 papers. Following data deduplication, 624 papers were excluded. Only 22 of the remaining 6023 papers matched our inclusion criteria and were included for full text screening. After critical appraisal we excluded five papers: three cohort studies papers presented a high risk of bias due to confounding, and one RCT paper presented a high risk of bias arising from the randomization process [21], [22], [23], [24] and one published preliminary results which were irrelevant to our research question [25] (see Appendix 2). Seventeen published papers fulfilling our inclusion criteria were included in this *meta*-analysis [2], [3], [9], [26], [27], [28], [29], [30], [31], [32], [33], [34], [35], [36], [37], [38], [39]. The PRISMA flow diagram is presented in Fig. 1.

We included six original RCTs, that were described in nine articles, with a total of 376 patients in the MIDCAB group and 376 patients in the PCI group [2], [3], [9], [34], [35], [36], [37], [38], [39]. Eight cohort studies were included with a total of 1283 patients in the MIDCAB group and 1812 patients in the PCI group [26], [27], [28], [29], [30], [31], [32], [33]. For both RCTs and cohort studies, follow-up varied from six months to ten years. The study design and characteristics of the studies included are summarized in Table 1.

**Table 1:** Summary of selected studies RCT.

Author, year of publication, country	Design RCT	Study period	Procedure	Population (N)	PCI (N)	MIDCAB (N)	Follow-up	Risk of bias
Diegeler et al. [2002] Germany Thiele et al. [2005] Germany Blazek et al. [2013] Germany	Open label single center randomized controlled trial	June 1997 – June 2001	MIDCAB under direct vision PCI with BMS	220	110	110	30 days 6-months 5 years 10 years	Low Low Low
Thiele et al. [2009] Germany Blazek et al. [2015] Germany	Open label single center randomized controlled trial	January 2003 – October 2007	MIDCAB under direct vision PCI with DES	130	65	65	30 days 1 year 7 years	Low Low
Cisowski et al. [2002] Poland	Open label single center randomized controlled trial	2000–2001	MIDCAB thoracoscopic assistance PCI with BMS	100	50	50	30 days 6-months 1 year	Low
Drenth et al. [2002] The Netherlands	Open label single center randomized controlled trial	March 1997 – September 1999	MIDCAB under direct vision PCI with BMS	102	51	51	30 days 6-months	Moderate
Kim et al. [2005] South Korea	Open label single center randomized controlled trial	January 2000 – December 2001	MIDCAB under direct vision and mini-sternotomy PCI with BMS	100	50	50	30 days 1 year	Low
Reeves et al. [2004] United Kingdom	Open label multicenter randomized controlled trial	November 1999 – December 2001	MIDCAB under direct vision or thoracoscopic assistance PCI with BMS	100	50	50	30 days 12 months	Low

Summary of selected cohort studies								
Author, year of publication, country	Design cohort	Study period	Procedure	Population (N)	PCI (N)	MIDCAB (N)	Follow-up	Risk of bias
Benedetto et al. [2014] United Kingdom	Retrospective single center prospective propensity score-matched comparison	April 2001 - May 2013	MIDCAB under direct vision or thoracoscopic assistance PCI with DES	1033 (before matching)	303	303	30 days 1 year 5 years 10 years	Moderate
Choi et al. [2019] South Korea	Retrospective single center prospective propensity score-matched comparison	September 2007 – June 2017	MIDCAB under direct vision PCI with DES	154	77	77	3 years	Moderate
Etienne et al. [2013] Belgium	Retrospective multicenter study prospective propensity score-matched comparison	1997–2011	MIDCAB under direct vision PCI with DES	456	196	260	30 days 5 years	Moderate
Iakovou et al. [2002] United States of America	Retrospective single center prospective propensity score-matched comparison	June 1996 – December 1999	MIDCAB under direct vision PCI with BMS	560	441	119	30 days 1 year	Moderate
Li et al. [2021] China	Retrospective single center prospective propensity score-matched comparison	July 2007 – November 2011	RA-MIDCAB PCI with DES	719 (before matching)	108	108	4 years	Moderate
Merkle et al. [2019] Germany	Retrospective single center matched comparison	2006–2012	MIDCAB harvesting under direct vision or using thoracoscopic assistance PCI with DES	206	100	106	1 year 6 years 10 years	Moderate
Shirai et al. [2004] United States of America	Retrospective single center matched comparison	February 1990 – October 1999	MIDCAB unknown technique PCI with BMS	581	429	152	30 days 6 months	Moderate
Patel et al. [2020] United States of America	Retrospective single center prospective propensity score-matched comparison	January 2008 –December 2016	RA-MIDCAB PCI with DES	531 (before matching)	158	158	9 years	Moderate

Abbreviations: BMS: bare metal stent, DES: drug eluting stent, RA-MIDCAB: robotic assisted minimally invasive direct coronary artery bypass, PCI: percutaneous coronary intervention, RCT: randomized controlled trial.

## 4 Study outcomes

### 4.1 Primary outcome

#### 4.1.1 All-cause mortality RCT and cohort studies

**Fig. 2:**
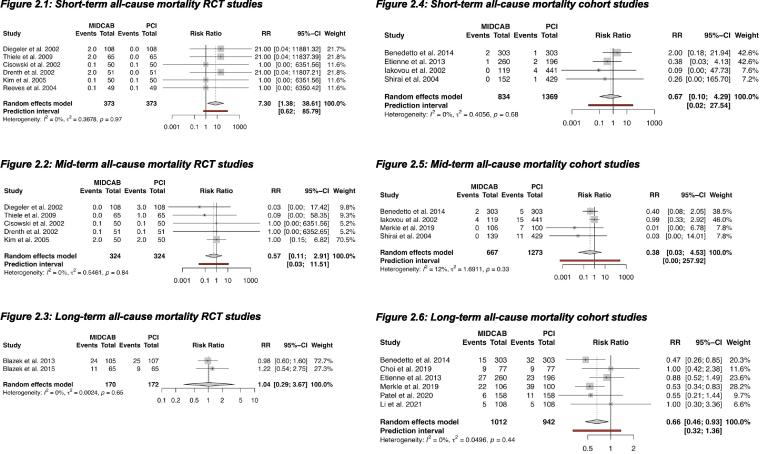
All-cause mortality. RR <1 is in favor of MIDCAB. Legend: Forest plots, short-term, mid-term and long-term all-cause mortality in RCTs and cohort studies. CI: confidence interval, MIDCAB: minimally invasive direct coronary artery bypass, PCI: percutaneous coronary interventions, RCT: randomized controlled trial, RR: risk ratio.

At short-term follow-up, all six RCTs showed a higher all-cause mortality in the MIDCAB group (RR 7.30, 95% CI: 1.38 to 38.61) (NNT = 100) [9], [34], [36], [37], [38], [39]. Five RCTs reported mid-term all-cause mortality and two trials reported long-term all-cause mortality [2], [3], [9], [34], [36], [37], [38], [39] with no difference in these 2 follow-up periods. (Fig. 2).

Four cohort studies reported short-term and mid-term all-cause mortality, with no difference between the two intervention groups [26], [28], [29], [31], [33]. At long-term follow-up, a benefit of MIDCAB over PCI was demonstrated (RR 0.66, 95% CI: 0.46 to 0.93) (NNT = 25) (Fig. 2) [26], [27], [28], [30], [31], [32].

## 5 Secondary outcomes

### 5.1 Cardiac mortality RCT and cohort studies

Six RCTs showed an increase in cardiac mortality after MIDCAB compared to PCI at short-term follow-up, which was significant (RR 7.30, 95% CI: 1.38 to 38.61) (NNT = 100) [9], [34], [36], [37], [38], [39]. At mid-term and long-term follow-up, no difference in cardiac mortality was found [9], [34], [36], [37], [38].

Short-term and long-term cardiac mortality were reported in two cohort studies [28], [29], [32] but we could not draw any conclusions because of the low numbers of events [2], [3]. No cohort studies reported mid-term cardiac mortality (Appendix 3).

### 5.2 rTVR RCT and cohort studies

In the RCTs at short-term no difference was found in rTVR between MIDCAB and PCI [9], [34], [36], [37], [38]. At mid-term follow-up a significant benefit of MIDCAB over PCI was demonstrated (RR 0.16, 95% CI: 0.11 to 0.23) (NNT = 10) [9], [34], [36], [37], [38]. At long-term follow-up only two RCTs reported rTVR which resulted in unstable estimates because of the low numbers of events [2], [3].

**Fig. 3:**
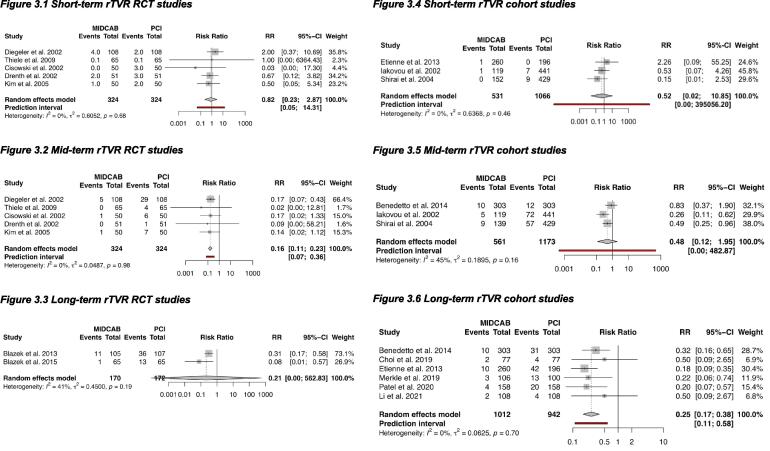
Repeat target vessel revascularization. RR <1 is in favor of MIDCAB. Legend: Forest plots, short-term, mid-term and long-term cardiac mortality in RCTs and cohort studies. CI: confidence interval, MIDCAB: minimally invasive direct coronary artery bypass, PCI: percutaneous coronary interventions, RCT: randomized controlled trial, RR: risk ratio, rTVR: repeat vessel revascularization.

In the cohort studies at short- and mid-term follow-up no difference was found in rTVR [28], [29], [33]. At long-term follow-up a decreased rTVR in the MIDCAB group over the PCI group was found (RR 0.25, 95% CI: 0.17 to 0.38) (NNT = 13) (Fig. 3) [26], [27], [28], [30], [31], [32].

### 5.3 MI RCT and cohort studies

No difference in MI was found at short-term, mid-term or long-term follow-up in both RCTs and cohort studies. (Appendix 4) [9], [28], [29], [33], [34], [36], [37], [38], [39].

### 5.4 CVA RCTs and cohort studies

No differences in CVAs was found in either the RCTs or the cohort studies when comparing MIDCAB and PCI (Appendix 5) [26], [27], [28], [29], [30], [33], [37], [38].

### 5.5 Heterogeneity and bias

Funnel plot asymmetry could be visually suspected in the RCTs for all-cause mortality and cardiac mortality at short-term and mid-term follow-up. Egger’s test also detected possible publication bias for these outcomes. In the cohort group funnel plot asymmetry was found for all-cause mortality at mid-term follow-up and Egger’s test detected publication bias (Appendix 6).

Analysing outcomes using fixed effects models showed no significant difference in pooled effect estimates when compared to the random-effects’ models.

Heterogeneity was considered as *I^2^* > 50% and moderate heterogeneity was present at mid-term follow-up rTVR in the RCT group.

## 6 Discussion

Our *meta*-analysis comparing MIDCAB with PCI suggests that for isolated lesions of the LAD, MIDCAB had a higher mortality risk at short-term follow-up. Short-term all-cause mortality was fully driven by cardiac mortality. At long-term follow-up, MIDCAB showed a survival benefit in the cohort studies. In addition, MIDCAB decreased rTVR at mid-term and long-term follow-up. No difference between MIDCAB and PCI in terms of MI and CVA risk was found.

Previous *meta*-analyses reported MIDCAB and PCI being both effective strategies for isolated LAD stenosis. They have shown similar clinical outcomes, but did not report a survival benefit in the MIDCAB group at long-term follow-up even though a decrease in mortality could be expected based on the proven survival benefit of the LITA-LAD conduit in conventional CABG [4], [40], [41]. We did find differences in mortality between the two treatments. In the RCTs we found at short-term follow-up an increase in all-cause mortality, which was fully driven by cardiac causes, in favor of PCI. Nevertheless, these results showed possible publication bias as assessed by Egger’s test and a wide confidence interval. The increased 30-day mortality after MIDCAB might be driven by the more invasive character of the procedure, the early-stage technique and little experience available at the time of the RCTs.

At long-term follow-up, the cohort studies showed a decreased all-cause mortality for MIDCAB when compared to PCI. This was not present in the RCTs, perhaps because long-term all-cause mortality was reported for only 2 RCTs with a small sample-size. Alternatively the difference may be caused by the moderate selection bias in the cohort studies (Appendix 2). However, an all-cause mortality benefit of conventional CABG over PCI has been confirmed by the SYNTAX trial for patients with a high (≥32) and intermediate (23–32) SYNTAX-score. The presence of the LITA graft has shown to be an independent predictor of survival and contributes significantly to superior long-term survival [10], [12], [13]. The LITA graft produces nitric oxide, inducing a vasodilator response in LAD protecting against atherosclerosis and thereby prevents MI and cardiac death in the long term [43]. Moreover, most patients undergoing MIDCAB have low SYNTAX-score (0–22). Hence, we expect the potential survival benefit of the use of the LITA also in patients receiving MIDCAB. This might explain the long-term survival of MIDCAB in the cohort studies.

In our analysis we confirmed that MIDCAB offers a decreased TVR at mid-term and long-term follow-up. MI and CVA rates were not different between MIDCAB and PCI, even though there was only limited experience with MIDCAB when the RCTs and cohort studies were conducted. We hypothesize that with increasing surgeons’ experience in this technique, fewer complications such as these will occur.

Only a small number of studies reported cardiac mortality, myocardial infarction and CVA. Several *meta*-analyses reported the incidence of composite outcomes such as Major Adverse Cardiac (and Cerebrovascular) Events (MAC(C)E). We excluded these as an outcome because of the variety of definitions for MAC(C)E used. We recommended the adoption of standard definition to allow adequate comparisons of future results.

The most recent European Guidelines for myocardial revascularization classified both CABG and PCI as class I, level A evidence for the management of proximal LAD disease [1]. However the optimal revascularization strategy for coronary artery disease is under constant debate because of the continuing development of surgical techniques and stent technology. PCI techniques have improved over the last years moving from BMS to third-generation DES. Secondary cardiovascular management changed with the introduction of more effective anti-thrombotic medications for better stent protection [44], [45], [46], [47]. In the past two decades, the adoption of MIDCAB for isolated proximal LAD lesions or in combination with PCI (hybrid coronary revascularization) increased worldwide. Nowadays LITA robotic-assisted harvesting induces minimal tissue damage optimizing the operation quality and reducing complications [42], [48], [49], [50]. Moreover, CABG and PCI differ substantially in revascularization mechanisms. CABG provides alternative vascularization routes addressing existing and future atherosclerotic lesions. PCI, in contrast, treats only existing lesions. Therefore, it has been observed that only CABG increases long-term survival in patients with stable coronary artery disease by providing “surgical collateralization” [51], [52], [53]. Our analysis confirmed long-term survival benefit in the MIDCAB group, though we do not know whether this is the result of lower cardiac mortality or of MI.

We acknowledge that this study has some limitations. Firstly, the number of included patients and the number of events were small across all studies. Secondly, moderate risk of bias was detected in all the cohort studies and possible publication bias was found in the primary outcome at short- and mid-term follow-up. The detected bias in the cohort studies was mainly because of selection and confounding. Selection bias could have distorted the published estimates of the articles. Our stated RR might therefore be over- or underestimated when it comes to the reported outcomes, according to the direction of distortion due to bias. The same applies for publication bias, being a type of selection bias. The possible presence of residual confounding in the included studies could have resulted in the unstable estimates pooled, hence the wide confidence intervals. Thus, our demonstrated short- and long-term outcomes should be interpreted with caution, unlike the more robust outcomes at long-term follow-up. Thirdly, we did not include considerations on LAD anatomy and its potential influence on SYNTAX-scores. Furthermore, MIDCAB is a technically demanding procedure and has therefore a long learning curve. We did not correct for differences between surgeons’ and centres expertise. Finally, because of a limited number of studies, we were not able to differentiate between LITA harvesting techniques or between different stents.

## 7 Conclusion

We did a *meta*-analysis of evidence from the past 20 years to compare MIDCAB with PCI in patients with proximal LAD lesions. The RCT data suggested that MIDCAB was associated with a higher short-term mortality, although a level A evidence, these analyses may be limited by possible publication bias. In contrast, in the cohort studies, a level B evidence, MIDCAB appeared to offer a long-term survival benefit. A decreased mid-term rTVR was demonstrated by the RCTs and cohort studies showed a decrease in rTVR rates in the long term. MIDCAB might therefore be considered an adequate first treatment option for proximal isolated LAD disease in selected patients. Multicenter RCTs with long-term follow-up that have adequate statistical power are required to confirm these results and to investigate if increased experience with MIDCAB has reduced the associated short-term mortality.

## Declaration of Competing Interest

The authors declare that they have no known competing financial interests or personal relationships that could have appeared to influence the work reported in this paper.
