# Supplementary material for: Minimally invasive surgery or stenting for left anterior descending artery disease – meta-analysis
Source: Int J Cardiol Heart Vasc. 2022 May 10;40:101046. doi: 10.1016/j.ijcha.2022.101046 (PMC9098394; doi:10.1016/j.ijcha.2022.101046)
Supplement: Supplementary data 1 [file mmc1.docx]

**Appendix 1. Search Strategy**

| *Search MEDLINE via Pubmed* |
| --- |
| ("Coronary Artery revascularization/methods"[Mesh] OR "Coronary Artery Bypass/methods"[Mesh] OR "Coronary Artery Disease/surgery"[Mesh] OR "Single coronary Artery Disease/surgery"[Mesh] OR "Internal Mammary-Coronary Artery Anastomosis"[Mesh] OR “left anterior descending artery”[tiab] OR “left main coronary artery”[tiab] OR “left main disease”[tiab] OR ”ramus descending anterior”[tiab] OR “LIMA-LAD”[tiab] OR “LITA-LAD”[tiab] OR “LIMA to LAD”[tiab] OR “LITA to LAD”[tiab] OR “LIMA on LAD”[tiab] OR “LITA on LAD”[tiab])) |
| **AND** |
| ("Minimally Invasive Surgical Procedures"[Mesh] OR “minimal* invasive* coronary arter* bypass graft*”[Mesh] OR "Robotic Surgical Procedures"[Mesh] OR “minimally invasive internal thoracic artery bypass”[tiab] OR “minimally invasive internal mammary artery bypass”[tiab] OR “MIDCAB”[tiab] OR “MINI-CAB”[tiab] OR “port-access”[tiab] OR “minimally invasive direct coronary artery bypass*”[tiab] OR “minimally invasive coronary artery bypass”[tiab] OR “RA-MIDCAB”[tiab] OR “Robot assist* MIDCAB”[tiab] OR “total* endoscopic coronary arter* bypass graft*”[tiab] OR “TECAB”[tiab]) |
| **AND** |
| ("Percutaneous Coronary Intervention"[Mesh] OR "Angioplasty, Balloon, Coronary"[Mesh] OR "Stents"[Mesh] OR "Drug-Eluting Stents"[Mesh] OR "Self Expandable Metallic Stents"[Mesh] OR “percutaneous coronary intervention”[tiab] OR PCI [tiab] OR “percutaneous coronary arter* stent*”[tiab] OR “percutaneous coronary revascularization”[tiab] OR “coronary balloon angioplasty”[tiab] OR “transluminal coronary balloon dilation”[tiab] OR “percutaneous transluminal coronary angioplasty”[tiab] OR  “PTCA” [tiab] OR “drug eluting stent*”[tiab] OR “drug-coated stent*”[tiab] OR “DES”[tiab] OR “coronary stent*”[tiab] OR “primary stent*”[tiab]) |

| *Search Embase* |
| --- |
| ('coronary artery surgery'/exp OR 'coronary artery atherosclerosis'/exp OR 'heart muscle revascularization'/exp OR 'left anterior descending coronary artery':ab,ti OR ‘left main coronary artery’:ab,ti OR ‘left main disease’:ab,ti OR ‘ramus descending anterior’:ab,ti OR ‘LIMA-LAD’:ab,ti OR ‘LITA-LAD’:ab,ti OR “LIMA to LAD’:ab,ti OR ‘LITA to LAD’:ab,ti OR ‘LIMA on LAD’:ab,ti OR “LITA on LAD’:ab,ti) |
| **AND** |
| ('minimally invasive cardiac surgery'/exp OR 'minimally invasive procedure'/exp OR 'minimally invasive surgery'/exp OR 'robotic surgical procedure'/exp OR 'robot assisted surgery'/exp OR ‘minimally invasive internal thoracic artery bypass’:ab,ti OR ‘minimally invasive internal mammary artery bypass’:ab,ti OR ‘MIDCAB’:ab,ti OR ‘MINI-CAB’:ab,ti OR ‘port-access’:ab,ti OR ‘minimally invasive direct coronary artery bypass*’:ab,ti OR ‘minimally invasive coronary artery bypass’:ab,ti OR ‘RA-MIDCAB’:ab,ti OR ‘Robot assist* MIDCAB’:ab,ti OR ‘total* endoscopic coronary arter* bypass graft*’:ab,ti OR ‘TECAB’:ab,ti) |
| **AND** |
| ('percutaneous coronary intervention'/exp OR 'percutaneous transluminal angioplasty'/exp OR 'stent'/exp OR 'drug eluting stent'/exp OR 'self expandable metallic stent'/exp OR ‘percutaneous coronary intervention’:ab,ti OR PCI:ab,ti OR ‘percutaneous coronary arter* stent*’:ab,ti OR ‘percutaneous coronary revascularization’:ab,ti OR ‘coronary balloon angioplasty’:ab,ti OR ‘transluminal coronary balloon dilation’:ab,ti OR ‘percutaneous transluminal coronary angioplasty’:ab,ti OR  ‘PTCA’:ab,ti OR ‘drug eluting stent*’:ab,ti OR ‘drug-coated stent*’:ab,ti OR ‘DES’:ab,ti OR ‘coronary stent*’:ab,ti OR ‘primary stent*’:ab,ti) |

| *Search Cochrane* |
| --- |
| (“left anterior descending artery” OR “left main coronary artery” OR “left main disease” OR ”ramus descending anterior” OR “LIMA-LAD” OR “LITA-LAD” OR “LIMA to LAD” OR “LITA to LAD” OR “LIMA on LAD” OR “LITA on LAD”) |
| **AND** |
| (“minimally invasive internal thoracic artery bypass” OR “minimally invasive internal mammary artery bypass” OR “MIDCAB” OR “MINI-CAB” OR “port-access” OR “minimally invasive direct coronary artery bypass*” OR “minimally invasive coronary artery bypass” OR “RA-MIDCAB” OR “Robot assist* MIDCAB” OR “total* endoscopic coronary arter* bypass graft*” OR “TECAB”) |
| **AND** |
| (“percutaneous coronary intervention” OR PCI OR “percutaneous coronary arter* stent*” OR “percutaneous coronary revascularization” OR “coronary balloon angioplasty” OR “transluminal coronary balloon dilation” OR “percutaneous transluminal coronary angioplasty” OR  “PTCA” OR “drug eluting stent*” OR “drug-coated stent*” OR “DES” OR “coronary stent*” OR “primary stent*”) |
