# Supplementary material for: Minimally invasive surgery or stenting for left anterior descending artery disease – meta-analysis
Source: Int J Cardiol Heart Vasc. 2022 May 10;40:101046. doi: 10.1016/j.ijcha.2022.101046 (PMC9098394; doi:10.1016/j.ijcha.2022.101046)
Supplement: Supplementary data 2 [file mmc2.docx]

**Appendix 2. Cochrane Risk of Bias Tool RCT and non-RCT – Table of Quality assessment**

| **Individual studies non-RCT**  **Bias category** | Ben-Gal et al. 2006 | Benedetto et al. 2014 | Choi et al. 2019 | Etienne et al. 2013 | Forouzandeh et al. 2018 | Fraund et al. 2005 | Iakovou et al. 2002 | Li et al. 2021 | Merkle et al. 2019 | Patel et al. 2020 | Shirai et al. 2004 |
| --- | --- | --- | --- | --- | --- | --- | --- | --- | --- | --- | --- |
| Bias due to confounding | + | +/- | +/- | +/- | + | + | +/- | +/- | +/- | +/- | +/- |
| Bias in selection of participants | +/- | +/- | +/- | +/- | +/- | +/- | +/- | +/- | +/- | +/- | +/- |
| Bias in classification of interventions | + | + | + | + | + | + | + | + | + | + | + |
| Bias due to deviations from intended interventions | + | + | + | + | + | + | + | + | + | + | + |
| Bias due to missing data | + | + | + | + | + | + | + | + | + | + | + |
| Bias in measurement of outcomes | + | + | + | + | + | + | + | + | + | + | + |
| Bias in selection of the reported result | + | + | + | + | + | + | + | + | + | + | + |
| **Overall quality** | High risk of bias | Moderate risk of bias | Moderate risk of bias | Moderate risk of bias | High risk of bias | High risk of bias | Moderate risk of bias | Moderate risk of bias | Moderate risk of bias | Moderate risk of bias | Moderate risk of bias |

| **Individual studies RCT**  **Bias category** | Diegeler et al. 2002  Thiele et al. 2005  Blazek et al. 2013 | Thiele et al. 2009  Blazek et al. 2015 | Cisowski et al. 2002 | Drenth et al. 2002 | Hong et al. 2005 | Kim et al. 2005 | Reeves et al. 2004 |
| --- | --- | --- | --- | --- | --- | --- | --- |
| Bias arising from the randomization process | + | + | + | +/- | - | + | + |
| Bias due to deviations from the intended interventions | + | + | + | +/- | + | + | + |
| Bias due to missing outcome data | + | + | + | + | + | + | + |
| Bias in measurement of the outcome | + | + | + | + | + | + | + |
| Bias in selection of the reported result | + | + | + | + | + | + | + |
| **Overall quality** | Low risk | Low risk | Low risk | Some concerns | High risk of bias | Low risk | Low risk |

Abbreviations: RCT: randomized controlled trial.
