# Supplementary material for: Minimally invasive surgery or stenting for left anterior descending artery disease – meta-analysis
Source: Int J Cardiol Heart Vasc. 2022 May 10;40:101046. doi: 10.1016/j.ijcha.2022.101046 (PMC9098394; doi:10.1016/j.ijcha.2022.101046)

Appendix 3.1 Short-term cardiac mortality RCT studies

Study	MIDCAB Events	MIDCAB Total	PCI Events	PCI Total	RR	95%-CI	Weight
Diegeler et al. 2002	2.0	108	0.0	108	21.00	[0.04; 11881.32]	21.7%
Thiele et al. 2009	2.0	65	0.0	65	21.00	[0.04; 11837.39]	21.8%
Cisowski et al. 2002	0.1	50	0.1	50	1.00	[0.00; 6351.56]	11.6%
Drenth et al. 2002	2.0	51	0.0	51	21.00	[0.04; 11807.21]	21.8%
Kim et al. 2005	0.1	50	0.1	50	1.00	[0.00; 6351.56]	11.6%
Reeves et al. 2004	0.1	49	0.1	49	1.00	[0.00; 6350.42]	11.6%
**Random effects model**		**373**		**373**	**7.30**	**[1.38; 38.61]**	**100.0%**
**Prediction interval**						**[0.62; 85.79]**	

Heterogeneity: $I^2 = 0\%$, $\tau^2 = 0.3678$, $p = 0.97$

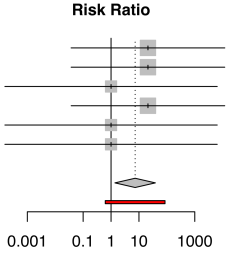

Appendix 3.2 Mid-term cardiac mortality RCT studies

Study	MIDCAB Events	MIDCAB Total	PCI Events	PCI Total	RR	95%-CI	Weight
Diegeler et al. 2002	0.1	108	0.1	108	1.00	[0.00; 6381.55]	16.9%
Thiele et al. 2009	0.1	65	0.1	65	1.00	[0.00; 6364.43]	17.0%
Cisowski et al. 2002	0.1	50	0.1	50	1.00	[0.00; 6351.56]	17.0%
Drenth et al. 2002	0.1	51	0.1	51	1.00	[0.00; 6352.65]	17.0%
Kim et al. 2005	0.0	50	2.0	50	0.05	[0.00; 26.77]	32.2%
**Random effects model**		**324**		**324**	**0.38**	**[0.05; 2.70]**	**100.0%**
**Prediction interval**						**[0.03; 5.43]**	

Heterogeneity: $I^2 = 0\%$, $\tau^2 = 0.1989$, $p = 0.96$

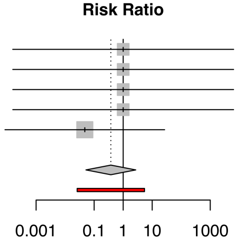

Appendix 3.3 Long-term cardiac mortality RCT studies

Study	MIDCAB Events	MIDCAB Total	PCI Events	PCI Total	RR	95%-CI	Weight
Blazek et al. 2013	10	105	9	107	1.13	[0.48; 2.67]	86.0%
Blazek et al. 2015	1	65	2	65	0.50	[0.05; 5.38]	14.0%
**Random effects model**		**170**		**172**	**1.01**	**[0.03; 37.08]**	**100.0%**

Heterogeneity: $I^2 = 0\%$, $\tau^2 = 0.0559$, $p = 0.53$

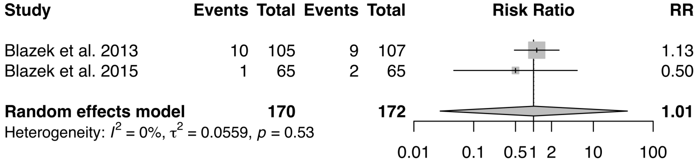

Appendix 3.4 Short-term cardiac mortality cohort studies

Study	MIDCAB Events	MIDCAB Total	PCI Events	PCI Total	RR	95%-CI	Weight
Etienne et al. 2013	0.1	260	0.1	196	0.75	[0; 4820.81]	35.3%
Shirai et al. 2004	0.0	152	1.0	429	0.26	[0; 165.70]	64.7%
**Random effects model**		**412**		**625**	**0.38**	**[0; 261.56]**	**100.0%**

Heterogeneity: $I^2 = 0\%$, $\tau^2 = 0.0107$, $p = 0.85$

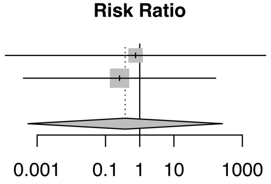

Appendix 3.5 Long-term cardiac mortality cohort studies

Study	MIDCAB Events	MIDCAB Total	PCI Events	PCI Total	RR	95%-CI	Weight
Choi et al. 2019	6	77	7	77	0.86	[0.30; 2.43]	58.2%
Etienne et al. 2013	3	260	7	196	0.32	[0.08; 1.23]	41.8%
**Random effects model**		**337**		**273**	**0.57**	**[0.00; 257.98]**	**100.0%**

Heterogeneity: $I^2 = 21\%$, $\tau^2 = 0.1847$, $p = 0.26$

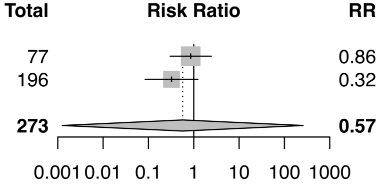

Supplement: Supplementary data 3 [file mmc3.pdf]
