# Supplementary material for: Minimally invasive surgery or stenting for left anterior descending artery disease – meta-analysis
Source: Int J Cardiol Heart Vasc. 2022 May 10;40:101046. doi: 10.1016/j.ijcha.2022.101046 (PMC9098394; doi:10.1016/j.ijcha.2022.101046)

Appendix 4.1 Short-term myocardial infarction RCT studies

Study	MIDCAB Events	MIDCAB Total	PCI Events	PCI Total	RR	95%–CI	Weight
Diegeler et al. 2002	4.0	108	2.0	108	2.00	[0.37; 10.69]	28.6%
Thiele et al. 2009	5.0	65	1.0	65	5.00	[0.60; 41.63]	22.6%
Cisowski et al. 2002	0.0	50	1.0	50	0.09	[0.00; 58.19]	4.0%
Drenth et al. 2002	1.0	51	5.0	51	0.20	[0.02; 1.65]	22.7%
Kim et al. 2005	1.0	50	2.0	50	0.50	[0.05; 5.34]	19.8%
Reeves et al. 2004	0.1	49	0.1	49	1.00	[0.00; 6350.42]	2.3%
**Random effects model**		**373**		**373**	**0.96**	**[0.22; 4.19]**	**100.0%**
**Prediction interval**						**[0.04; 21.27]**	

Heterogeneity: $I^2 = 16\%$, $\tau^2 = 0.9149$, $p = 0.31$

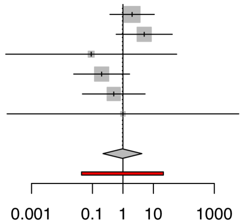

Appendix 4.2 Mid-term myocardial infarction RCT studies

Study	MIDCAB Events	MIDCAB Total	PCI Events	PCI Total	RR	95%–CI	Weight
Diegeler et al. 2002	1.0	108	1.0	108	1.00	[0.06; 15.78]	61.3%
Thiele et al. 2009	0.1	65	0.1	65	1.00	[0.00; 6364.43]	6.6%
Cisowski et al. 2002	0.1	50	0.1	50	1.00	[0.00; 6351.56]	6.6%
Drenth et al. 2002	0.1	51	0.1	51	1.00	[0.00; 6352.65]	6.6%
Kim et al. 2005	0.1	50	0.1	50	1.00	[0.00; 6351.56]	6.6%
Reeves et al. 2004	0.0	50	2.0	50	0.05	[0.00; 26.77]	12.5%
**Random effects model**		**374**		**374**	**0.68**	**[0.22; 2.17]**	**100.0%**
**Prediction interval**						**[0.12; 3.75]**	

Heterogeneity: $I^2 = 0\%$, $\tau^2 = 0.1736$, $p = 0.98$

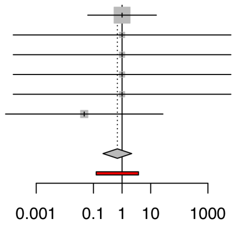

Appendix 4.3 Long-term myocardial infarction RCT studies

Study	MIDCAB Events	MIDCAB Total	PCI Events	PCI Total	RR	95%–CI	Weight
Blazek et al. 2013	12	105	5	107	2.45	[0.89; 6.70]	52.1%
Blazek et al. 2015	6	65	6	65	1.00	[0.34; 2.94]	47.9%
**Random effects model**		**170**		**172**	**1.59**	**[0.01; 465.37]**	**100.0%**

Heterogeneity: $I^2 = 29\%$, $\tau^2 = 0.1654$, $p = 0.23$

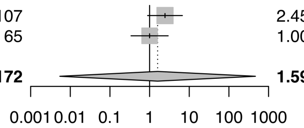

Appendix 4.4 Short-term myocardial infarction cohort studies

Study	MIDCAB Events	MIDCAB Total	PCI Events	PCI Total	RR	95%–CI	Weight
Etienne et al. 2013	1	260	4	196	0.19	[0.02; 1.67]	59.8%
Iakovou et al. 2002	0	119	2	441	0.18	[0.00; 100.02]	20.4%
Shirai et al. 2004	1	152	0	429	31.03	[0.05; 20049.36]	19.8%
**Random effects model**		**531**		**1066**	**0.51**	**[0.00; 254.22]**	**100.0%**
**Prediction interval**						**[0.00; 6317653800513.03]**	

Heterogeneity: $I^2 = 8\%$, $\tau^2 = 3.5460$, $p = 0.34$

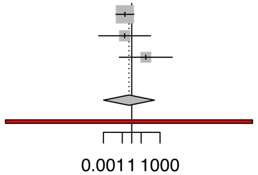

Appendix 4.5 Mid-term myocardial infarction cohort studies

Study	MIDCAB Events	MIDCAB Total	PCI Events	PCI Total	RR	95%–CI	Weight
Iakovou et al. 2002	0	119	3	441	0.12	[0.00; 64.66]	9.6%
Shirai et al. 2004	1	139	11	429	0.28	[0.04; 2.15]	90.4%
**Random effects model**		**258**		**870**	**0.26**	**[0.01; 6.29]**	**100.0%**

Heterogeneity: $I^2 = 0\%$, $\tau^2 = 0.0113$, $p = 0.80$

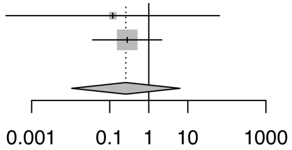

Appendix 4.6 Long-term myocardial infarction cohort studies

Study	MIDCAB Events	MIDCAB Total	PCI Events	PCI Total	RR	95%–CI	Weight
Choi et al. 2019	2	77	3	77	0.67	[0.11; 3.88]	61.7%
Li et al. 2021	1	108	3	108	0.33	[0.04; 3.15]	38.3%
**Random effects model**		**185**		**185**	**0.51**	**[0.01; 36.99]**	**100.0%**

Heterogeneity: $I^2 = 0\%$, $\tau^2 = 0.0244$, $p = 0.63$

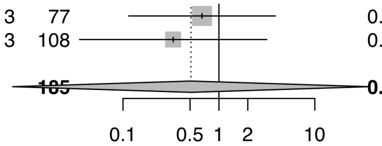

Supplement: Supplementary data 4 [file mmc4.pdf]
