# Supplementary material for: Minimally invasive surgery or stenting for left anterior descending artery disease – meta-analysis
Source: Int J Cardiol Heart Vasc. 2022 May 10;40:101046. doi: 10.1016/j.ijcha.2022.101046 (PMC9098394; doi:10.1016/j.ijcha.2022.101046)

Appendix 5.1 Mid-term CVA RCT studies

Study	MIDCAB Events	MIDCAB Total	PCI Events	PCI Total	RR	95%−CI	Weight
Drenth et al. 2002	0	51	1	51	0.09	[0.00; 58.21]	50.0%
Kim et al. 2005	1	50	0	50	11.00	[0.02; 7041.58]	50.0%
**Random effects model**		**101**		**101**	**1.00**	**[0.00; 17067741612823.66]**	**100.0%**

Heterogeneity: $I^2 = 5\%$, $\tau^2 = 3.9786$, $p = 0.30$

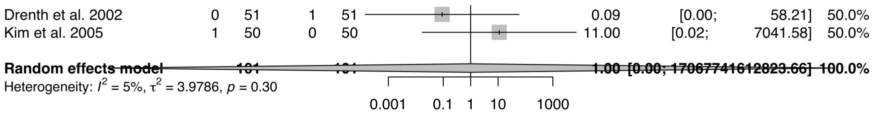

Appendix 5.2 Short-term CVA cohort studies

Study	MIDCAB Events	MIDCAB Total	PCI Events	PCI Total	RR	95%−CI	Weight
Benedetto et al. 2014	0.1	303	0.1	303	1.00	[0.00; 6398.24]	4.1%
Etienne et al. 2013	0.0	260	2.0	196	0.04	[0.00; 20.37]	7.6%
Iakovou et al. 2002	1.0	119	6.0	441	0.62	[0.08; 5.08]	48.0%
Shirai et al. 2004	1.0	152	2.0	429	1.41	[0.13; 15.45]	40.3%
**Random effects model**		**834**		**1369**	**0.71**	**[0.13; 3.97]**	**100.0%**
**Prediction interval**						**[0.01; 40.56]**	

Heterogeneity: $I^2 = 0\%$, $\tau^2 = 0.5917$, $p = 0.76$

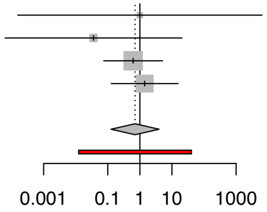

Appendix 5.3 Mid-term CVA cohort studies

Study	MIDCAB Events	MIDCAB Total	PCI Events	PCI Total	RR	95%−CI
Shirai et al. 2004	2	139	10	429	0.62	[0.14; 2.78]

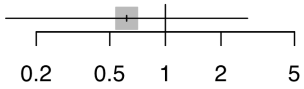

Appendix 5.4 Long-term CVA cohort studies

Study	MIDCAB Events	MIDCAB Total	PCI Events	PCI Total	RR	95%−CI	Weight
Choi et al. 2019	0	77	1	77	0.09	[0.00; 58.44]	2.8%
Etienne et al. 2013	4	260	5	196	0.60	[0.16; 2.22]	41.4%
Li et al. 2021	8	108	7	108	1.14	[0.43; 3.04]	55.8%
**Random effects model**		**445**		**381**	**0.82**	**[0.19; 3.56]**	**100.0%**
**Prediction interval**						**[0.00; 3106.54]**	

Heterogeneity: $I^2 = 0\%$, $\tau^2 = 0.3038$, $p = 0.59$

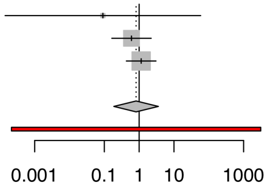

Supplement: Supplementary data 5 [file mmc5.pdf]
