# Supplementary material for: Minimally invasive surgery or stenting for left anterior descending artery disease – meta-analysis
Source: Int J Cardiol Heart Vasc. 2022 May 10;40:101046. doi: 10.1016/j.ijcha.2022.101046 (PMC9098394; doi:10.1016/j.ijcha.2022.101046)

Appendix 6.1: Short-term all-cause mortality FP RCT

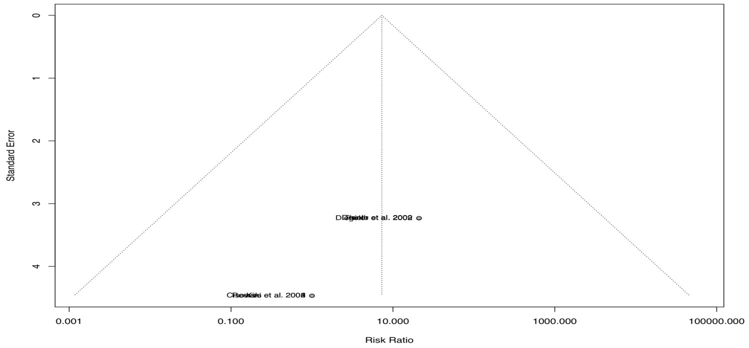

Appendix 6.2: Mid-term all-cause mortality FP RCT

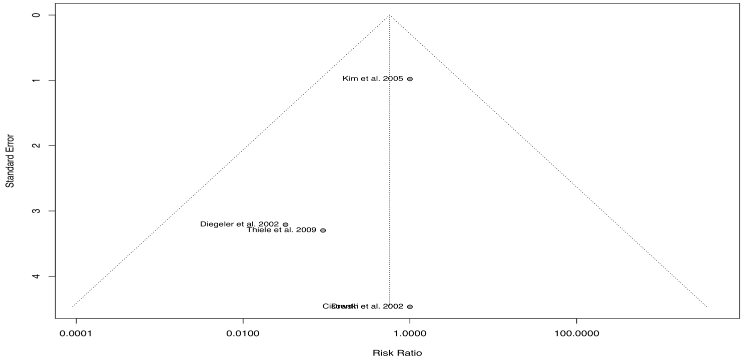

Appendix 6.3: Long-term all-cause mortality FP RCT

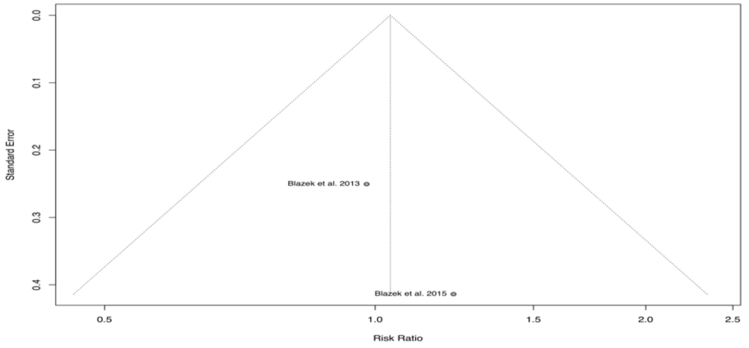

Appendix 6.4: Short-term all-cause mortality FP cohort studies

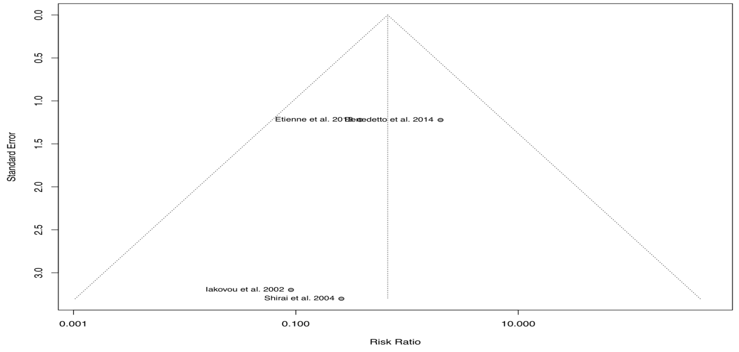

Appendix 6.5: Mid-term all-cause mortality FP cohort studies

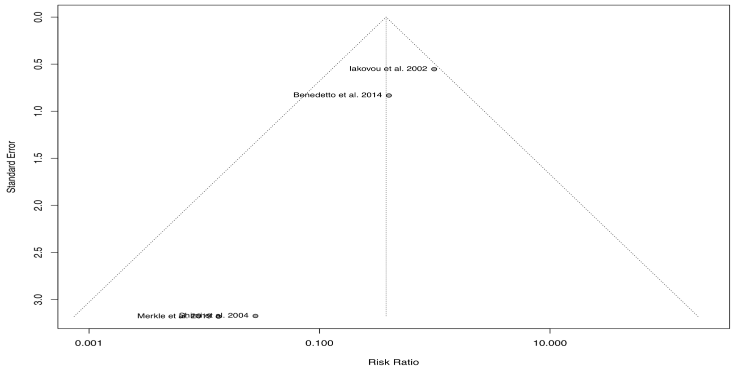

Appendix 6.6: Long-term all-cause mortality FP cohort studies

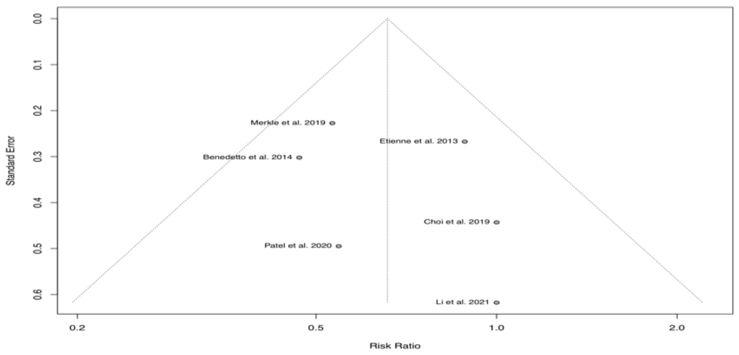

Appendix 6.7: Short-term cardiac mortality FP RCT

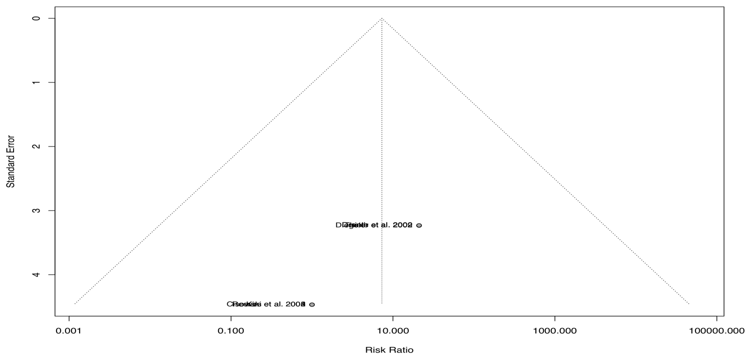

Appendix 6.8: Mid-term cardiac mortality FP RCT

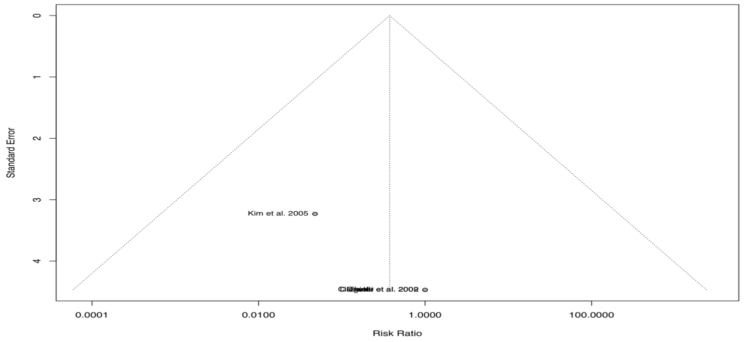

Appendix 6.9: Long-term cardiac mortality FP RCT

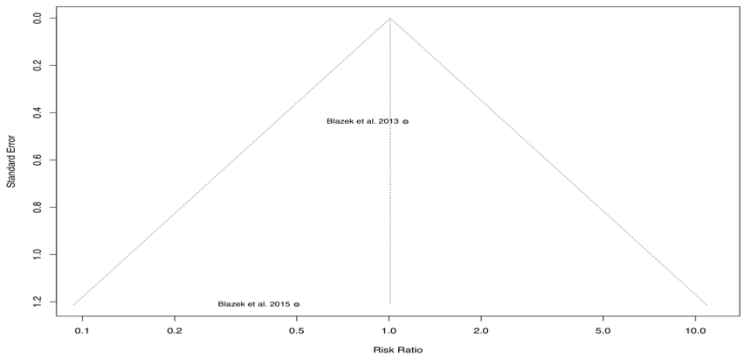

Appendix 6.10: Short-term cardiac mortality FP cohort studies

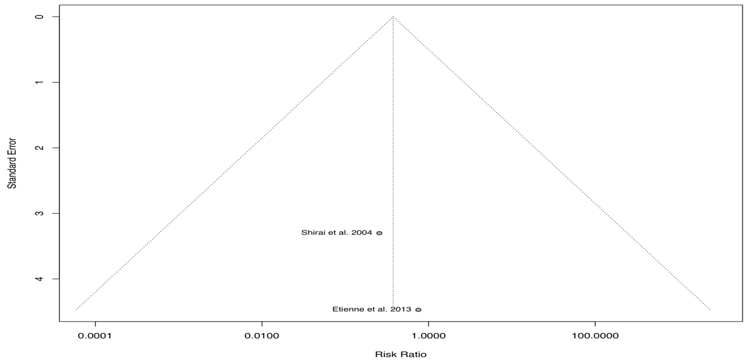

Appendix 6.11: Long-term cardiac mortality FP cohort studies

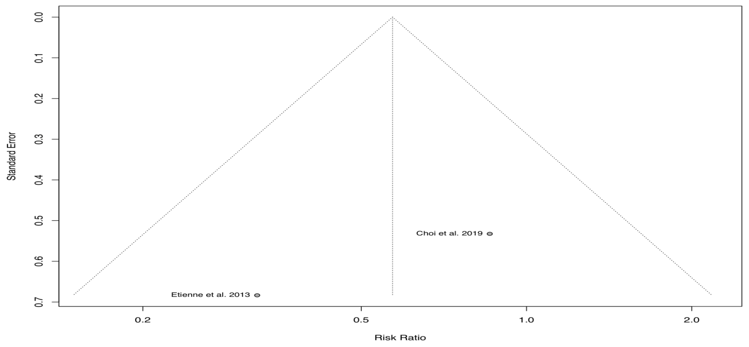

Appendix 6.12: Short-term TVR FP RCT

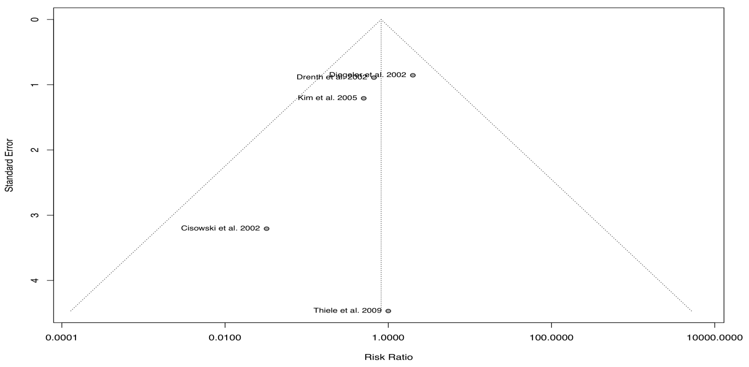

Appendix 6.13: Mid-term TVR FP RCT

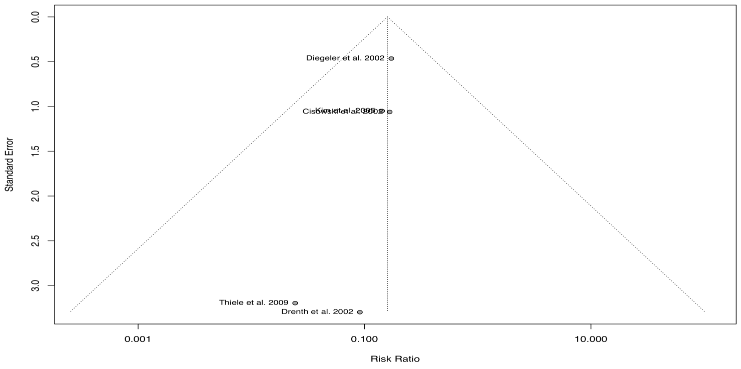

Appendix 6.14: Long-term TVR FP RCT

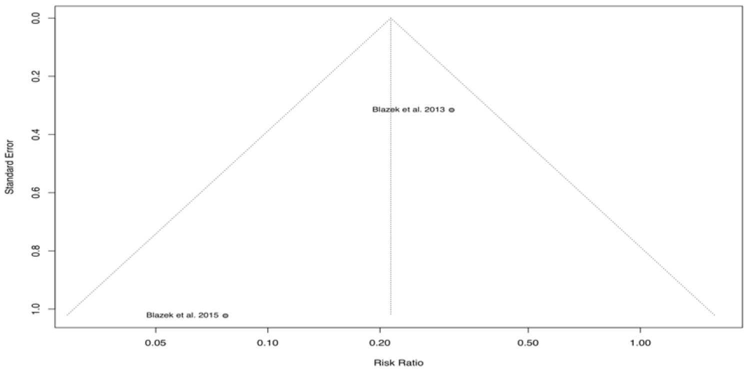

Appendix 6.15: Short-term TVR FP cohort studies

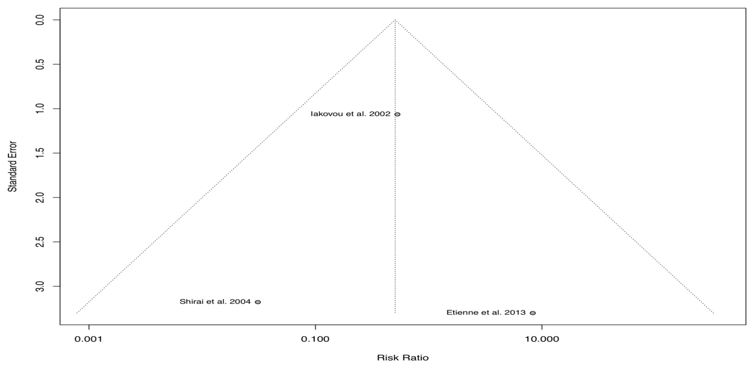

Appendix 6.16: Mid-term TVR FP cohort studies

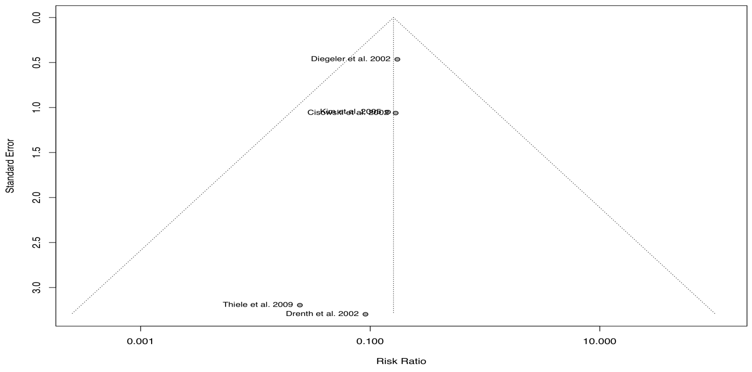

Appendix 6.17: Long-term TVR FP cohort studies

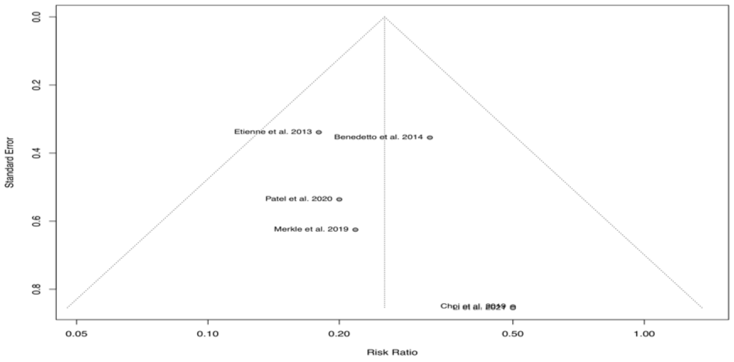

Appendix 6.18: Short-term MI FP RCT

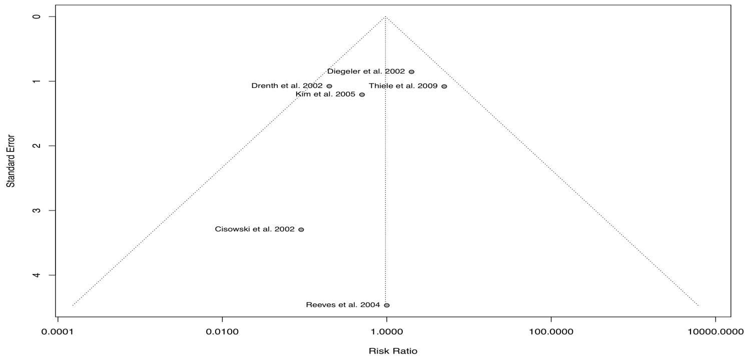

Appendix 6.19: Mid-term MI FP RCT

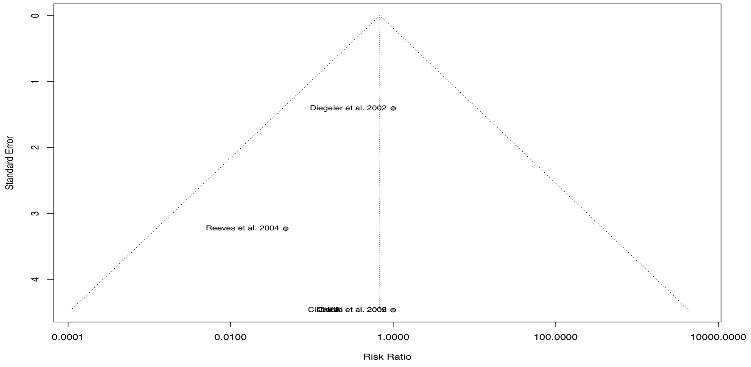

Appendix 6.20: Long-term MI FP RCT

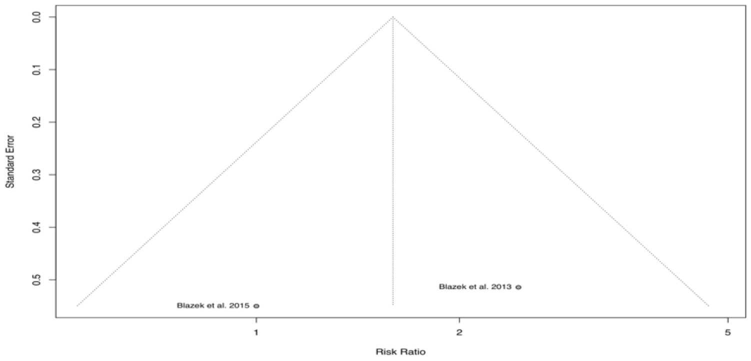

Appendix 6.21: Short-term MI FP cohort studies

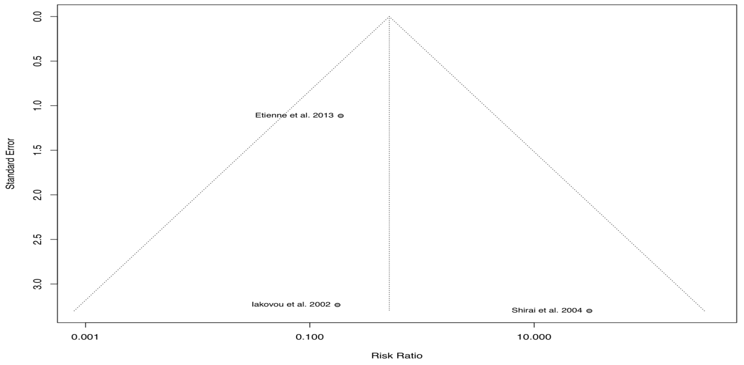

Appendix 6.22: Mid-term MI FP cohort studies

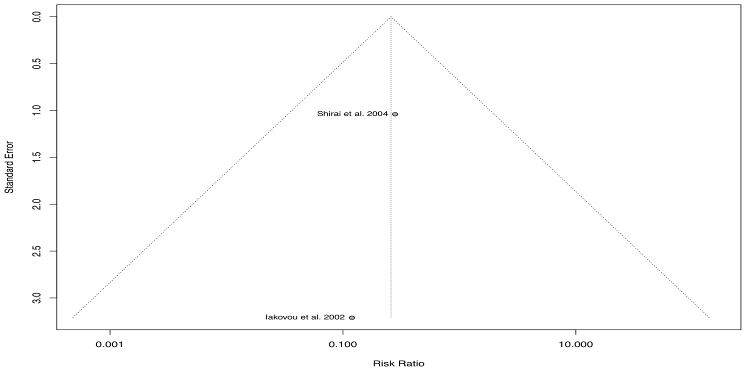

Appendix 6.23: Long-term MI FP cohort studies

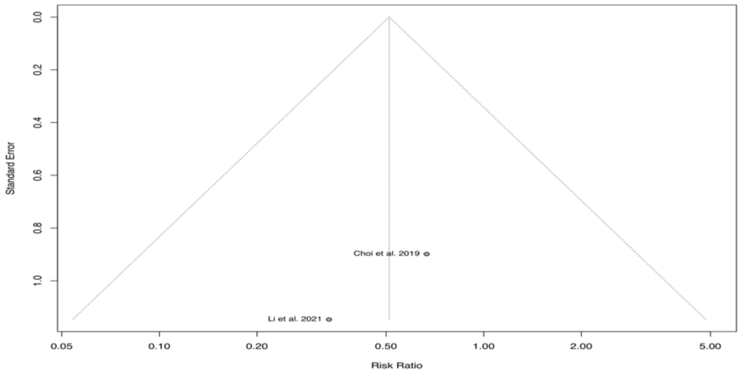

Appendix 6.24: Mid-term CVA FP RCT

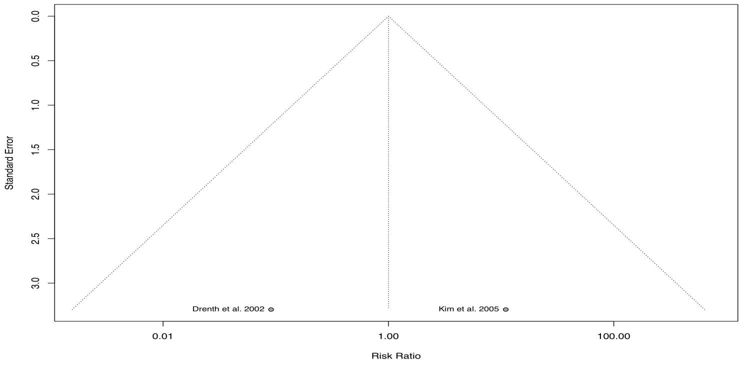

Appendix 6.25: Short-term CVA FP cohort studies

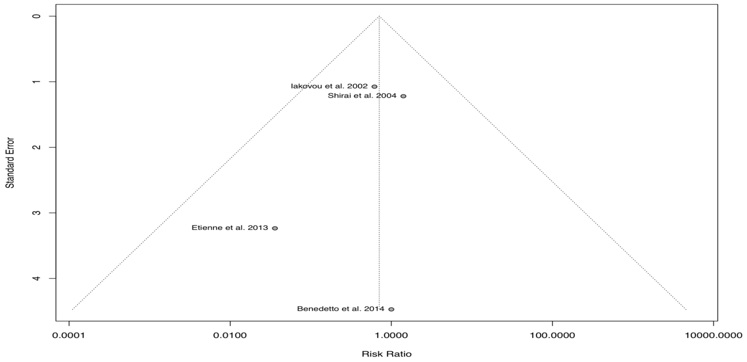

Appendix 6.26: Mid-term CVA FP cohort studies

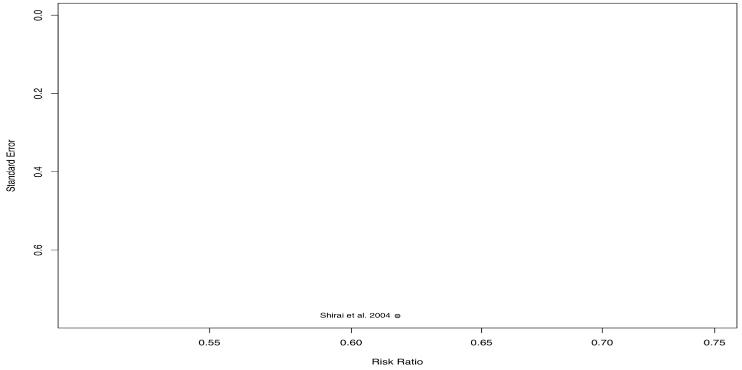

Appendix 6.27: Long-term CVA FP cohort studies

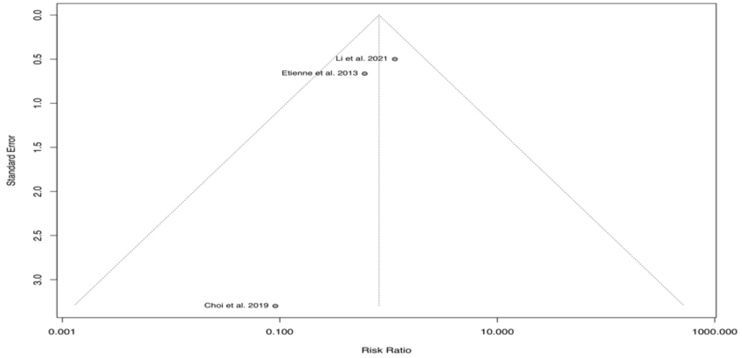

Supplement: Supplementary data 6 [file mmc6.pdf]
